# Case Report: Prolonged nOPV2 shedding in an immunocompetent child from Tunisia border with Algeria, North African region

**DOI:** 10.3389/fmed.2026.1904434

**Published:** 2026-07-22

**Authors:** Sondes Haddad-Boubaker, Haifa Khemiri, Wasfi Fares, Najla Mekki, Chaima Alibi, Imen Ben Salem, Henda Touzi, Zina Meddeb, Ines Ben Mrad, Mahrez Yahyaoui, Javier Martin, Imen Ben Mustapha, Ahlem Gzara, Henda Triki

**Affiliations:** 1 Laboratory of Clinical Virology, WHO Regional Reference Laboratory for Poliomyelitis and Measles for in the Eastern Mediterranean Region, Institut Pasteur de Tunis, University of Tunis El Manar, Tunis, Tunisia; 2 Research Laboratory “Viruses, Vectors and Hosts” (LR20IPT02), Institut Pasteur de Tunis, University of Tunis El Manar, Tunis, Tunisia; 3 Laboratory of Transmission, Control and Immunobiology of Infection (LR 11 IPT 02), Institut Pasteur de Tunis, University of Tunis El Manar, Tunis, Tunisia; 4 Faculty of Medicine, University of Tunis El Manar, Tunis, Tunisia; 5 Basic Health Care Directorate, Ministry of Health Tunis, Tunis, Tunisia; 6 National Institute for Biological Standards and Control, South Mimms, United Kingdom

**Keywords:** complete genome, cVDPV2, kinetics, nomadic, nOPV2, prolonged excretion

## Abstract

Following the detection of vaccine-derived poliovirus type 2 (cVDPV2) in Algeria in 2022, enhanced surveillance activities along the Tunisian border identified novel Oral Poliovirus type 2 Vaccine (nOPV2) excretion in an apparently healthy nomadic child. Immunological investigations showed no evidence of major humoral, cellular, or phagocytic immune deficiency. To investigate the kinetics of viral shedding and characterize the complete genome of the isolated strain in this case report, longitudinal follow-up stool samples were collected and analyzed using cell culture inoculation, real-time RT-PCRs, and whole-genome sequencing. Virus was detected over a 76 to 126-days observation period. Assuming vaccination occurred in December 2022, the total duration of shedding may have extended up to approximately 138 to 188 days (4 to 7 months) post-vaccination. Despite prolonged replication, the viruses showed limited within-host evolution and retained the major engineered attenuation features of nOPV2, with no evidence of domain V reversion, cre replacement, or recombination, and only limited VP1 divergence which allows its classification as nOPV2 category 8 strains. Our findings underscore the need to further investigate the potential for prolonged excretion and molecular evolution of nOPV2-like strains in immunocompetent individuals. Furthermore, targeted surveillance of high-risk and mobile populations, including migrants and nomadic communities, is essential to strengthen epidemiological security and support global poliovirus eradication initiatives.

## 1 Introduction

The trivalent Oral Poliovirus Vaccine (tOPV), developed in late 1950 by Albert Sabin, is a major tool in global poliovirus eradication efforts. It contains live attenuated strains of the three poliovirus serotypes (PV1, PV2, and PV3) (1). This vaccine contributed significantly to a major milestone in the fight against polio allowing global eradication of wild poliovirus type 2 (WPV2) and type 3 (WPV3). in September 2015 and 2019, respectively (2, 3). Nevertheless, genetic evolution of live attenuated strains leads to the emergence of vaccine-derived polioviruses (VDPV), mainly type 2, responsible for multiple outbreaks worldwide (4, 5). In the aim to reduce the risk of VDPV, type 2-containing OPV was withdrawn worldwide in April 2016 (6) and was replaced by the bivalent OPV (bOPV) (2, 3).

Notably, by the end of 2020, outbreaks of cVDPV2 re-emerged in parts of Africa, Europe, and the Middle East, 64 cVDPV2 outbreaks had been reported, resulting in 1,572 cases of paralytic polio. The use of monovalent OPV2 (mOPV2), for outbreak response, may have contributed to the persistence of VDPV2 circulation (2).

In response, the WHO Global Polio Eradication Initiative (GPEI) launched a new global strategy. Since March 2021, the novel Oral Poliovirus Vaccine type 2 (nOPV2), a genetically modified version of the Sabin vaccine, has been deployed under the WHO Emergency Use Listing (EUL) (7). The nOPV2 strain incorporates genetic modifications designed to enhance safety, efficacy, and genetic stability. They targeted key regions of the genome: the cis-acting replication element (cre) in the 5′ non-coding region, the “S15” domain V, and the RNA-dependent RNA polymerase (7, 8). Compared to Sabin2, nOPV2 has demonstrated a 75–80% reduction in the emergence of VDPV (7). By June 2022, only 2% of all whole-genome sequenced nOPV2 isolates showed mutations in domain V, the primary attenuation site of the vaccine virus, associated with public health consequences (7). Several studies have reported that nOPV2 can be excreted in stool samples for up to 124 days following vaccination in children (9–13).

On July 8, 2022, the first cVDPV2 case emerged in Tamanrasset province, southern Algeria (14, 15). Genomic sequencing revealed that the isolated virus was genetically related to a strain previously identified in Kano, Nigeria (NIE-ZAS-1) (16). In response, the Algerian Ministry of Health launched a comprehensive strategy focused on intensive vaccination using nOPV2 and enhanced surveillance (14, 17).

In Tunisia, following the detection of cVDPV2 in Algeria, enhanced surveillance activities were implemented in border areas, including stool sample collection from nomadic populations. Among the samples collected, one obtained from a healthy child tested positive for nOPV2. The child was subsequently monitored until viral clearance was confirmed.

In this study, we describe the kinetics of excretion and the molecular evolution of the obtained nOPV2 isolates through complete genome sequencing of all excreted isolates.

## 2 Materials and methods

### 2.1 Ethical statement

This study was approved by the Bio-Medical Ethics Committee of Pasteur Institute of Tunis, Tunisia (2019/20/I/LR161PT/V1). It was performed under ethical standards according to the 1964 Declaration of Helsinki and its later amendments. Written informed consent was provided by the parents or legal tutors of participants. The samples were collected as part of poliovirus (PV) surveillance activities and analyzed after de-identification to ensure patient anonymity.

### 2.2 Case description

An eight-year-old nomadic boy, originally from El-Oued governorate in Algeria and living between the two countries, tested positive for nOPV2 in February 2023 in Tunisia. The child had received a single dose of nOPV2 in Algeria in December 2022 (the exact day was not recorded) and subsequently travelled to the governorate of Tozeur, Tunisia. He had no familial or personal medical history including prior episodes of Acute Flaccid Paralysis or other neurological complications.

An immunological evaluation was performed, including a complete blood cell count, assessment of serum immunoglobulin level, immunophenotyping using a panel of cell surface markers, the nitroblue tetrazolium test, and lymphocyte proliferation assays.

The case was placed under close follow-up, which included monthly stool sample collection until virological clearance was confirmed. In total, five stool samples were collected between February 19 and June 25, 2023. Additionally, stool samples from six vaccinated contacts of the child, collected between February and June 2023, were obtained and revealed negative for nOPV2.

### 2.3 nOPV2 detection from stool samples

The stool samples were treated with PBS and chloroform and detected polio-positives by isolation on cell culture and intra-typic differentiation real-time RT-PCR (ITD), following the standard WHO protocols for poliovirus surveillance as previously described (5, 18–20).

### 2.4 Whole genome sequencing

The whole genome sequencing was carried out at the laboratory of Clinical Virology at Pasteur Institute of Tunis using Oxford Nanopore Technologies (ONT) (Oxford, UK). Viral RNA obtained from stool samples, was first reverse transcribed into complementary DNA (cDNA) using Luna^®^ Universal RT SuperMix (New England Biolabs, United States). The resulting cDNA was subsequently amplified using two pools of virus-specific primers designed with PrimalScheme (v3.0.2), generating overlapping amplicons of approximately 1,200 bp (Supplementary Table 1). Amplicons from both primer pools were combined and normalized to a total input of 50 ng for library preparation.

Sequencing libraries were prepared using NEBNext kit (New England Biolabs, USA), the Ligation Sequencing Kit (SQK-LSK109, ONT), and the Native Barcoding Expansion 1–12 Kit (EXP-NBD104, ONT), according to the manufacturers’ instructions.

### 2.5 Data analysis

Raw sequences generated from whole genome sequencing were analyzed using Geneious software (v2023.1.1). Data analysis was conducted using the “Map Reads then Find Variations/SNPs” workflow, with the *nOPV2* as reference genome (accession number MZ245455.1). This workflow integrates tools for sequence mapping using the Geneious Aligner, variant calling using the Geneious Variant Finder and consensus sequence generation using the built-in Geneious Aligner. The resulting consensus sequences were then submitted to NCBI; their corresponding accession numbers are provided: PZ398140-43. Recombination analysis was also performed using Geneious software (v2023.1.1) and Simplot software (v3.5.1).

## 3 Results

### 3.1 Case investigation

Immunological investigation of the case showed No lymphopenia and serum quantitative immunoglobulin levels (IgG 11.33 g/L, IgA 1.87 g/L and IgM 1.18 g/L) were within age-adjusted reference ranges. Lymphocyte immunophenotyping demonstrated normal T, B, and NK cell counts as well as normal percentage of recent thymic emigrants (RTE) lymphocytes. Expression of HLA class II molecules and nitroblue tetrazolium (NBT) test were both normal. Furthermore, lymphocyte proliferation in response to mitogens [phytohemagglutinin (PHA)] was comparable to healthy control. Accordingly, no evidence of major humoral, cellular, or phagocytic immune deficiency was identified using the investigations performed.

### 3.2 nOPV2 detection

**Figure 1:**
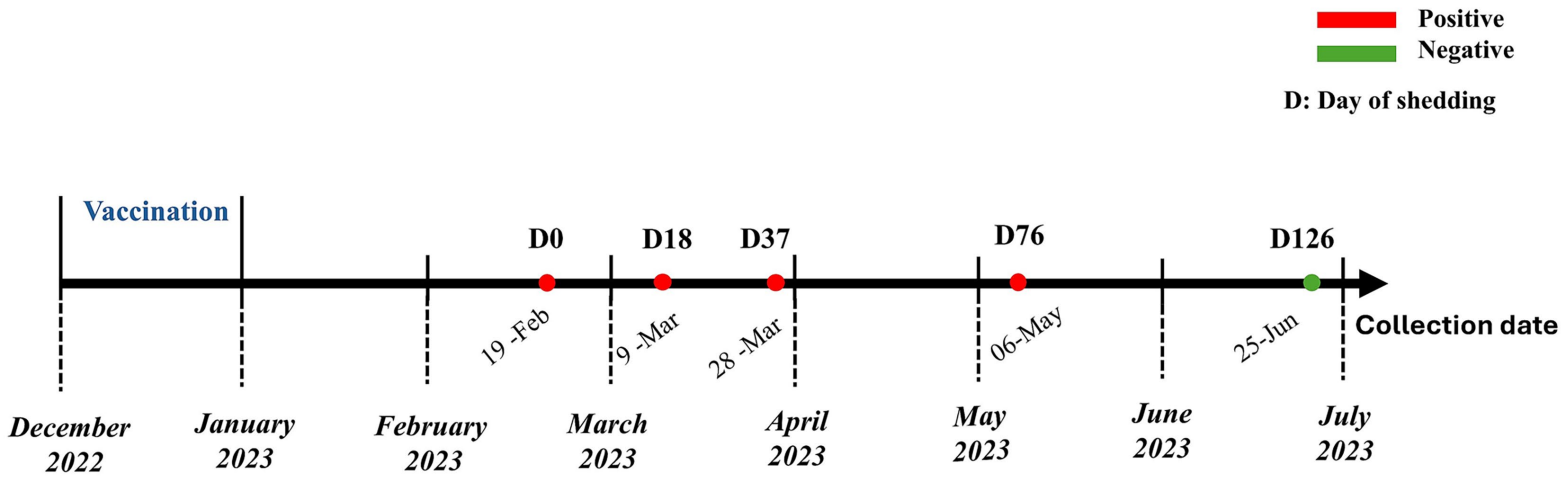
Kinetics of nOPV2 excretion in stool samples over time.

On the five stool samples collected from the investigated case, four tested positive for poliovirus based on the presence of a cytopathic effect (CPE) observed on both L20B and RD cell lines followed by confirmation using the WHO-recommended real-time RT-PCR assay. The first positive sample, collected on 19 February 2023, was detected approximately two months after the administration of the nOPV2 vaccine. By June 25, 2023, no virus was detected in the collected stool samples. Thus, viral excretion was observed from 19 February to 06 May 2023 and stopped by 25 June 2023, during a period ranged from 76 days to 126 days. Assuming vaccination occurred in December 2022, the total duration of shedding may have extended up to approximately 138–188 days post-vaccination (approximately 4 to 7 months) post-vaccination (Figure 1).

### 3.3 Whole genome sequencing

**Figure 2:**
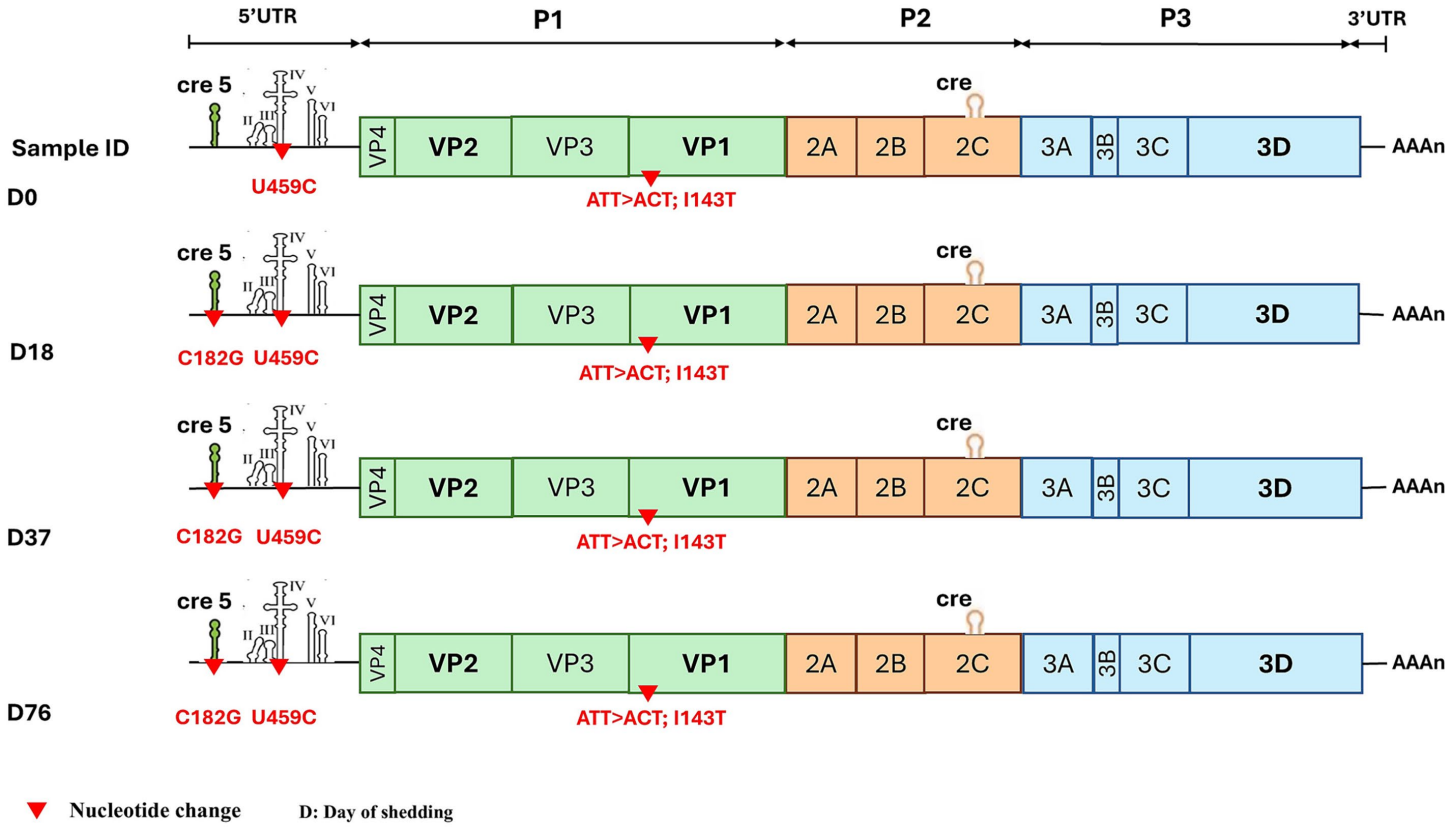
Nucleotide changes detected in each sequence of nOPV2.

Complete genome sequences were obtained for all four positive samples with 100% coverage. Sequence analysis identified two nucleotide changes in all samples (*n* = 4): U459C in domain IV, and an ATT < ACT substitution at positions 2,969–2,971 of the genome, corresponding to an I143T amino acid change in VP1. In addition, a nucleotide change (C182G) in the 5′ non-coding region was detected in three samples collected on days 18, 37 and 76 (Figure 2). Based on these findings, the isolate was classified as category 8 which corresponds to nOPV2-like viruses retaining the engineered attenuation features, without domain V reversion, cre replacement, or recombination, and with limited VP1 divergence (<6), as previously described by Martin (8).

## 4 Discussion

In the present study, we report unusually prolonged nOPV2 shedding, for approximately 7 months, considering the nOPV2 vaccination date, in a nomadic child, with no evidence of major humoral, cellular, or phagocytic immune deficiency. This child remained asymptomatic throughout the infection period, and all his contacts tested negative for nOPV2. Based on the whole-genome characterization, isolates showed limited genetic evolution and preserved nOPV2 attenuation features without any recombination events which allows its classification as category 8.

The nOPV2, a genetically modified version of the Sabin type 2 strain, was developed and introduced in several countries, in response to the ongoing cVDPV2 outbreaks. This vaccine was designed to enhance safety, immunogenicity, and genetic stability, particularly by stabilizing the primary attenuation site (domain V) to reduce the risk of reversion to neurovirulence (2). Evidence from clinical trials, primarily involving infants and young children, has demonstrated that nOPV2 is well tolerated, with safety outcomes (7). A phase 3 trial in Gambia involving 2,345 infants and 600 young children reported that 7 days post-dose one, 41.7% infants were excreting type 2 poliovirus, with no safety concerns identified (12). Similarly, in a phase 2 trial in Bangladesh, fecal shedding in newborns was monitored for 28 days following two doses of nOPV2, with no documented safety risks (10). Also, data obtained across six countries, during the initial use phase (March–October 2021), showed that the duration of nOPV2 excretion in stool samples ranged from 0 to 81 days (> 2 months) after vaccination (mean = 12.5 days; median = 7 days) (8, 21). Particularly, data from GPEI, about over 600 million doses administered across 28 countries confirm that nOPV2 shedding typically resolves within weeks in the vast majority of vaccinated, with the upper range of excretion (up to 124 days) representing rare outliers (9). Nevertheless, Ivanova et al. (11) further highlighted the possible prolonged excretion up to 124 days.

To the best of our knowledge, our finding constitutes the first report of prolonged excretion duration substantially exceeding the previously documented duration of 124 days (11) and reaching approximately 4 to 7 months post-vaccination with no underlying medical conditions. It’s worthy to note that while long replication period, isolated strains showed limited within-host evolution and retained the major engineered attenuation features of nOPV2, with no evidence of domain V reversion, cre replacement, or recombination, and with only limited VP1 divergence. Although these substitutions, including C182G at the base of cre5, U459C in domain IV, and VP1-I143T, are known to slightly reduce nOPV2 attenuation, the isolates retained the major determinants of the nOPV2 attenuated and genetically stable phenotype. Thus, these viruses were classified as category 8 viruses. This virus category represents the most frequently reported subgroup, accounting for 85–93.9% of isolates. Nevertheless, continuous genomic surveillance and vigilance are required since a smaller proportion of isolates have been classified as category 6 (2.6%) and category 9 (2.2%) (8, 22) and In addition, three isolates identified in Uganda were classified as category 2 and exhibited a double recombinant structure and (21). Given that nOPV2 was introduced under an Emergency Use Listing from March 2021, available data remain largely derived from controlled trials and initial surveillance phases with relatively short follow-up, which may underestimate the true upper limits of excretion in real-world populations. Therefore, continued systematic surveillance, including AFP case investigation, longitudinal follow-up of vaccinated individual and genomic sequencing, is crucial for fully characterizing the spectrum of nOPV2 excretion and genomic evolution, and to provide the additional evidence needed as vaccine deployment expands globally and key insights for the development of genetically modified type 1 and type 3 poliovirus vaccines. Ivanova et al. (11) further highlighted the importance of continued surveillance among migrant children from countries with documented polio outbreaks. Similarly, in our case, the nOPV2 prolonged excreter was detected through enhanced surveillance activities implemented in Tunisian border with Algeria, including stool sample collection from nomadic populations, after detection of cVDPV2 in Algeria. Nomadic, migrant and refugees’ populations often lack documented vaccination histories; such was the case in our study. Stool sampling is logistically challenging, especially when nomadic groups are mobile through difficult-to-access desert regions. Nevertheless, such studies are essential for detecting poliovirus introductions and implementing control measures in hard-to-reach populations. These findings also underscore the critical importance of strengthening global poliovirus surveillance, including both Acute Flaccid Paralysis (AFP) and environmental surveillance (ES), while maintaining high vaccination coverage. Such efforts are essential to detect and prevent silent poliovirus circulation and to mitigate the risk of importation and transmission of WPV and VDPV, even in countries with historically high vaccination coverage, such as Tunisia which is integrated with the AFP surveillance, Primary immunodeficient patient surveillance and ES, respectively, in 2021 and January 2026, in addition to the high maintained vaccination coverage.

## 5 Conclusion

In conclusion, we report prolonged excretion of nOPV2 following vaccination in a healthy child, without evidence of major humoral, cellular, or phagocytic immune deficiency. Although global surveillance data support the genetic stability of nOPV2, our study adds to the knowledge on the excretion kinetics and the molecular evolution of nOPV2 strains and highlights the need to further investigate the potential for prolonged excretion of nOPV2-like strains. Sustained genomic surveillance together with robust acute flaccid paralysis (AFP), environmental surveillance is essential to detect such events and to better understand the evolutionary dynamics and safety profile of nOPV2 and to provide key insights for the development of genetically modified type 1 and type 3 poliovirus vaccines.

## Data Availability

The datasets presented in this study can be found in online repositories. The names of the repository/repositories and accession number(s) can be found in the article/Supplementary material.
