# Supplementary material for: Case Report: Prolonged nOPV2 shedding in an immunocompetent child from Tunisia border with Algeria, North African region
Source: Front Med (Lausanne). 2026 Jul 22;13:1904434. doi: 10.3389/fmed.2026.1904434 (PMC13439408; doi:10.3389/fmed.2026.1904434)
Supplement: Supplementary file 1 [file Supplementary_File_1.docx]

Supplementary material

The sequences generated in this work are available at NCBI database under the following accession numbers:

PZ398140

PZ398141

PZ398142

PZ398143
